# Tissue immune profiles supporting response to mesenchymal stromal cell therapy in acute graft-versus-host disease—a gut feeling

**DOI:** 10.1186/s13287-019-1449-9

**Published:** 2019-11-20

**Authors:** Caroline Gavin, Erik Boberg, Lena Von Bahr, Matteo Bottai, Anton Törnqvist Andrén, Annika Wernerson, Lindsay C. Davies, Rachael V. Sugars, Katarina Le Blanc

**Affiliations:** 1 Department of Laboratory Medicine, Karolinska Institutet, Huddinge, Sweden; 2 Center of Hematology, Karolinska University Hospital, Stockholm, Sweden; 3 Department of Environmental Medicine, Unit of Biostatistics, Karolinska Institutet, Solna, Sweden; 4 Department of Pathology, Karolinska University Hospital, Huddinge, Sweden; 5 Department of Dental Medicine, Karolinska Institutet, Huddinge, Sweden; 6 Center for Allogeneic Stem Cell Transplantation, Karolinska University Hospital, Stockholm, Sweden

**Keywords:** Mesenchymal stromal cell, Acute graft-versus-host disease, Gut, Immunity, Adoptive cell therapy

## Abstract

Acute graft-versus-host disease (aGvHD), post-allogeneic hematopoietic stem cell transplantation, is associated with high mortality rates in patients not responding to standard line care with steroids. Adoptive mesenchymal stromal cell (MSC) therapy has been established in some countries as a second-line treatment.

Limitations in our understanding as to MSC mode of action and what segregates patient responders from non-responders to MSC therapy remain. The principal aim of this study was to evaluate the immune cell profile in gut biopsies of patients diagnosed with aGvHD and establish differences in baseline cellular composition between responders and non-responders to subsequent MSC therapy.

Our findings indicate that a pro-inflammatory immune profile within the gut at the point of MSC treatment may impede their therapeutic potential. These findings support the need for further validation in a larger cohort of patients and the development of improved biomarkers in predicting responsiveness to MSC therapy.

To the Editor:

Acute graft-versus-host disease (aGvHD) is the second leading cause of death, after disease relapse, in patients treated with allogeneic hematopoietic stem cell transplantation (aHSCT). aGvHD principally manifests in the skin, liver, and gastrointestinal (GI) tract, with approximately 60% of patients experiencing manifestation within the gut [1]. The disease arises due to recognition of patient antigens by the transplanted donor T cells, often symptomatically resulting in diarrhea, skin rash, and elevated bilirubin levels, with patients suffering from recurrent infections. aGvHD is graded according to severity from I–IV depending on the extent of organ involvement. Steroids are the first-line treatment but complete response only occurs in 35% of patients with steroid treatment alone [2]. Those who do not respond may advance to severe steroid-refractory aGvHD with high mortality, and limited standard second-line treatment options [3].

The role of T cells in promoting aGvHD pathophysiology has long been reported, with the CD8+ compartment postulated to be activated by the recipient’s hematopoietic APCs, whereas CD4+ cells can also be activated by non-hematopoietic APCs within the gut [3]. Activated antigen-presenting cells (APCs) present alloantigens to the donor T cells, with these cellular interactions driving the proliferation and differentiation of T cells, with T helper (Th)1 maturation strongly linked to GI tract pathology. Proliferating T cells differentiate and secrete a cocktail of factors, including interleukin (IL)-2 and interferon γ. IL-2 potentiates this pro-inflammatory cycle, by further activation of both T and natural killer (NK) cells, ultimately resulting in organ damage [4]. It is the loss in balance of suppressive regulatory T cells (Tregs) to effector CD4+ T cells and elevated NK cells that provide the hallmarks of aGvHD pathology [5].

Later studies have highlighted the importance of the intestinal epithelium and change in microbiome with aHSCT in GvHD pathogenesis [6]. The role of tissue damage, induced by conditioning regimens, has been re-evaluated with the knowledge that patients receiving donor lymphocyte infusions, where no conditioning regimen is utilized, are still afflicted by GvHD. aHSCT itself may cause damage to immune stem cells and Paneth cells, with the latter producing antimicrobial peptides that shape the microbiome of the GI tract, resulting in intestinal dysbiosis, potentiating aGvHD development.

Adoptive mesenchymal stromal cell (MSC) therapy has been investigated for the treatment of aGvHD due to their inherent immunosuppressive and immunomodulatory properties [7]. To date, bone marrow-derived MSCs have safely been utilized in numerous clinical trials to mitigate adverse immune and inflammatory diseases. MSCs have been documented to exert immunomodulatory effects primarily through contact-independent mechanisms. Despite low-level engraftment of transplanted MSCs, their ability to modulate both innate and adaptive immune responses has been documented, with long-term therapeutic effects on tolerance [8]. The primary mode of MSC action remains elusive, due to their plasticity and ability to respond according to microenvironmental changes. It is postulated that through balancing their suppressive and activating phenotype, these stromal cells can orchestrate immune and tissue repair responses [9]. It is for this reason that a clearer understanding into how the cellular microenvironment of MSC responder and non-responder patient cohorts differ is needed in order to improve therapeutic efficacy.

We have previously reported our phase II trial data, with 71% of steroid-resistant, severe aGvHD patients treated with MSCs responding to therapy [7]. While survival differs dramatically in complete responders to MSC therapy compared to non-responders, the underlying differences in GvHD biology are unknown [10]. The aim of this current study was to understand whether the composition of immune cells in the gut mucosa influenced responsiveness to MSC therapy. The findings suggest that a pro-inflammatory immune profile within the gut at the point of MSC treatment may impede therapeutic potential. These results demonstrate the need for further investigation into the role of the patient’s immunological milieu for responsiveness to MSC therapy.

A retrospective analysis was performed on gut mucosa biopsies taken for routine diagnostic purposes from suspected aGvHD patients presenting with diarrhea and abdominal pain, prior to MSC therapy (*n* = 16). Biopsies were taken from multiple sites within the colon. Ethical approval for research use of biopsy material was received from the local ethics committee, and included patients provided written consent in line with the Helsinki Declaration. All patients were later classified as having steroid-refractory aGvHD (defined as resistance to treatment with no overall improvement in GvHD grade or disease progression) [7] and received intravenous MSC therapy, of which 8 patients were deemed responders and 8 non-responders. Responder patients exhibited resolution of GvHD symptoms without additional treatment, and non-responders demonstrated no clinically evaluable response or progression of the disease. There were no statistical differences in terms of treatment, age, gender, and time from biopsy to MSC infusion between cohorts (Table 1). Histopathological grading according to Glucksberg criteria for aGvHD revealed similar global clinical classification grades III–IV between patients. Cytomegalovirus (CMV) colitis was detected in 2 responders and 3 non-responders to MSC therapy. No statistical difference in the prevalence of CMV colitis between the responder and non-responder groups was observed.

**Table 1:** Demographics of acute graph-versus-host disease patients receiving mesenchymal stromal cell therapy

Characteristic	Responders	Non-responders	*P* value
Total number of patients	8	8	
Age at HSCT (years): median (min–max)	58.4 (34.0–64.9)	54.3 (13.5–65.8)	0.96^a^
Female sex: *N* (%)	4 (50)	2 (25)	0.61^b^
Underlying disease: *N* (%)			
Myeloid neoplasm	4 (50)	7 (87.5)	0.35^b^
Lymphoid neoplasm	2 (25)	1 (12.5)	
Plasma cell dyscrasia	1 (12.5)	0 (0)	
Prostate cancer	1 (12.5)	0 (0)	
Donor: *N* (%)			
HLA-identical sibling	5 (62.5)	5 (62.5)	1^b^
Matched unrelated donor	3 (37.5)	3 (37.5)	
Conditioning: *N* (%)			
Standard	5 (62.5)	6 (75)	1^b^
Reduced intensity (RIC)	3 (37.5)	2 (25)	
Timeline (days): median (min–max)			
Time from HSCT or DLI to aGVHD	32.5 (13–107)	35.5 (11–169)	0.96a
Time from aGVHD diagnosis to steroid treatment	1.5 (0–7)	1 (0–7)	0.91^a^
Time from steroid treatment to MSC treatment	8 (3–44)	15 (4–55)	0.17^a^
Time from steroid treatment to biopsy^#^	2 (0–13)	6 (1–29)	0.18^a^
Biopsy before initiation of steroid treatment (*N*)	1	0	1^b^
Time from biopsy to MSC treatment	5.5 (2–37)	9.5 (3–26)	0.34^a^
aGVHD global clinical classification: *N* (%)			
Grades 0–I	0 (0)	0 (0)	NA
Grade II	0 (0)	0 (0)	
Grades III–IV	8 (100)	8 (100)	
GI aGVHD pathological classification: *N* (%)			
Grades 0–I	0 (0)	3 (37.5)	0.077^b^
Grade II	0 (0)	1 (12.5)	
Grades III–IV	8 (100)	4 (50)	
CMV infection: *N* (%)			
CMV colitis	2 (25)	3 (37.5)	1^b^
CMV viremia (> 1000 copies/ml)	2 (25)	4 (50)	0.61^b^
Leukocyte counts at time of biopsy: Mean (± SD) ^¶^			
Total leukocytes (× 10^9^)/L)	11.26 (± 1.86)	9.86 (± 8.28)	1^a^
Neutrophils (× 10^9^/L)	8.98 (± 1.81)	7.79 (± 6.54)	1^a^
Eosinophils (× 10^9^/L)	0.04 (± 0.07)	0.05 (± 0.09)	0.82^a^
Basophils (× 10^9^/L)	0.01 (± 0.01)	0.05 (± 0.09)	0.65^a^
Lymphocytes (× 10^9^/L)	0.90 (± 0.66)	0.63 (± 0.49)	0.60^a^
Monocytes (× 10^9^/L)	1.00 (± 0.70)	1.19 (± 1.47)	0.84^a^

^a^Wilcoxon rank-sum test

^b^Fisher’s exact test

^#^Excluding patients that were biopsied before steroid treatment

^¶^Samples taken > 6 days from date of biopsy are excluded

*GI* gastro-intestinal, *CMV* cytomegalovirus, *MSC* mesenchymal stromal cell, *aGVHD* acute graft-versus-host disease, *DLI* donor lymphocyte infusion, *HSCT* hematopoietic stem cell transplantation, *NA* not available, *aHSCT* allogeneic hematopoietic stem cell transplantation, *HLA* human leukocyte antigen, *RIC* reduced intensity conditioning, *DLI* donor lymphocyte infusion, *SEM* standard error of the mean

Immunohistochemical analysis for T cell subsets (CD4+, CD8+, and FoxP3+), mast cells (MCs; ß-tryptase), phagocytes (CD68+), and immunostimulatory CD56+ immune cells was performed at the Department of Pathology, Karolinska University Hospital, Huddinge, Sweden. These specific immune cell subsets were chosen for investigation based on the known pathophysiology of the disease, in addition to key innate immune populations implicated in MSC mode of action. One field of view (× 40 magnification, 1366 × 768 screen size = 683 × 706 dpi/image) was acquired per biopsy that covered the majority of intact tissue. Total chromogenic (3,3-diaminobenzidine; DAB) stained area per image total pixel area was quantified using CellProfiler software version 2 (https://cellprofiler.org/) (Additional file 1) [11]. Statistical analyses were performed using generalized estimated equations with the Poisson family (Stata version 14; StatCorp LLC, TX, USA).

Significantly higher CD8+ staining was detected in responders compared to non-responders (Fig. 1a; *P* ≤ 0.001). This corresponded with significantly lower levels of CD4+ T cells within the responder group (Fig. 1b; *P* ≤ 0.001). It could be postulated that the higher levels of CD8+ T cells prior to MSC therapy may provide a niche environment for the induction of CD8+CD28− Tregs, an immune subset previously correlated to clinical efficacy in chronic GvHD trials with MSCs, and promotion of allograft tolerance [12, 13]. The effect of MSCs on CD14+ monocytes in inducing their differentiation towards an anti-inflammatory, tolerogenic phenotype is well documented [9]. Furthermore, these MSC-primed monocytes have been reported to directly induce CD8+ Tregs, which in turn downregulate APC function by inducing immunoglobulin-like transcript 3 and 4 inhibitory receptors, culminating in the inhibition of proliferating CD4+ T cells linked to allograft rejection [13]. Additionally, significantly higher levels of FoxP3+ staining were seen in the responders compared to the non-responders (Fig. 1c; *P* ≤ 0.001). The transcription factor FoxP3 is primarily known for its role in Treg maturation, although it has also been demonstrated to exert other immunomodulatory and anti-inflammatory roles, as a negative regulator of conventional T cell (Tconv) proliferation and cytokine production, as well as, suppressing interferon γ production in Th17 cells [14].

**Fig. 1:**
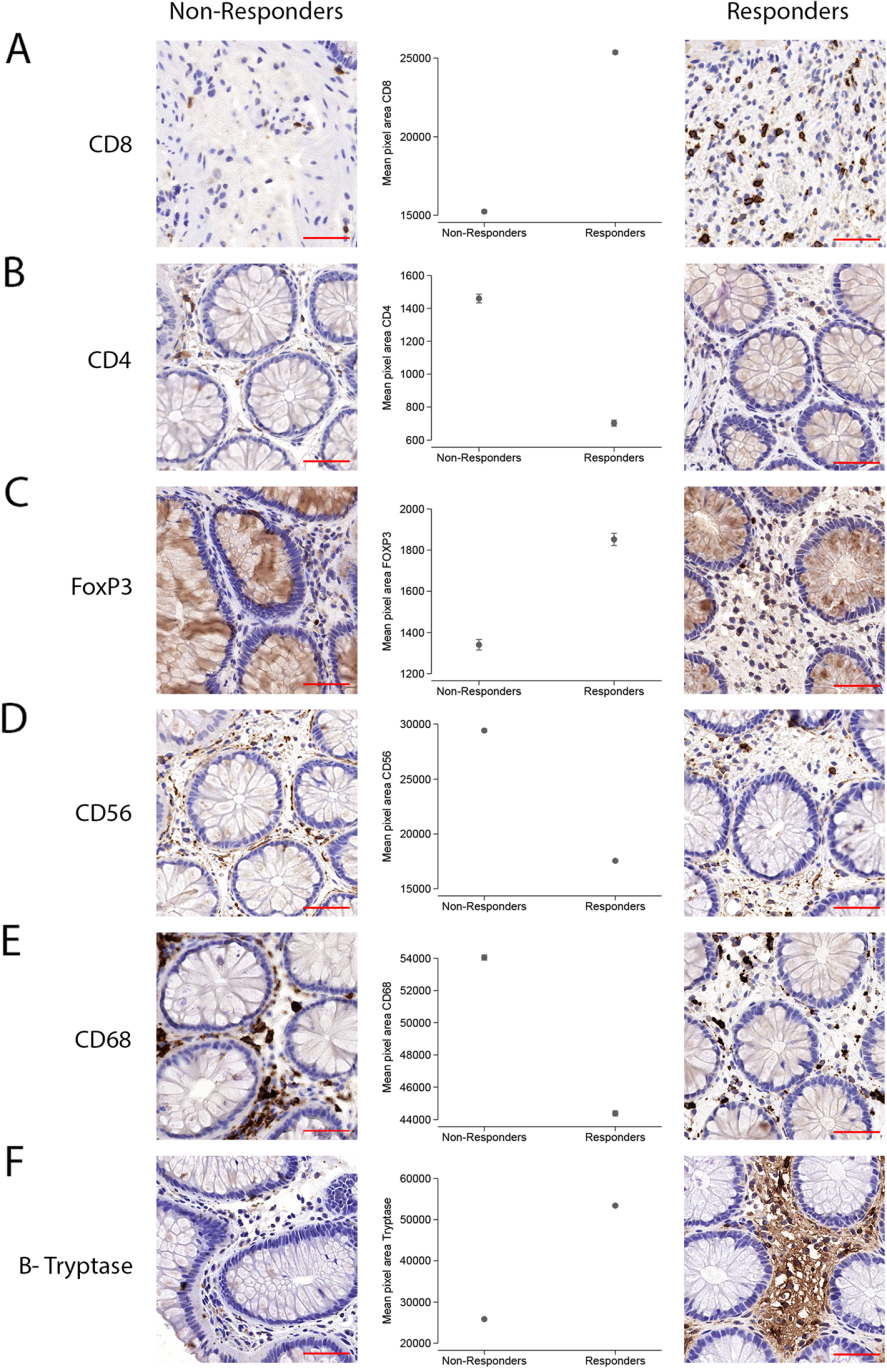
The tissue immune profile of the gut is distinct in non-responders to MSC therapy. Immunohistochemistry of gastrointestinal acute graft-versus-host disease (aGvHD) biopsies of responder and non-responder patients to mesenchymal stromal cell (MSC) therapy. Biopsies were taken after allogeneic hematopoietic stem cell transplantation (aHSCT), but prior to MSC infusion. Sections were immunohistochemically stained, with DAB, for antibodies targeted against **a** CD8, **b** CD4, **c** FoxP3, **d** CD56, **e** CD68, and **f** ß-tryptase. Corresponding graphs illustrate quantification of immunohistochemical staining from one high power field at × 40 magnification represented as mean pixel area (total DAB area stained/total image pixel area) with 95% confidence intervals (Additional file 1). Non-responders demonstrated an immune milieu suggestive of acute inflammation, potentially less supportive to MSC responsiveness, with significantly higher levels of staining for CD4+ T cells, CD56+ immunostimulatory cells, and CD68+ phagocytes. Scale bar = 50 μm

CD56 is a hallmark of NK and NK-T cells, but can also be found on the cell surface of other immune cells including monocytes and dendritic cells. Collectively, this glycoprotein is linked to immunostimulatory functions including Th1 cytokine production. CD56 staining (Fig. 1d; *P* ≤ 0.001), as well as the phagocytic marker, CD68 (Fig. 1e; *P* ≤ 0.001), expressed on immune cell subsets including monocytes, tissue-resident macrophages, and to a lesser extent dendritic cells, was both found to be elevated in the non-responder group. These parallel observations suggest a pro-inflammatory environment within the gut of non-responders and the need for a less acute inflammatory milieu in order for MSCs to be of therapeutic value.

MCs have been linked to reduction of gut GvHD in murine models through an IL-10-mediated suppression of Tconv proliferation, independent of Tregs [15]. Both tryptase-positive MC (MC_T_) and tryptase- and chymase-positive MC (MC_TC_) subsets of MCs express ß-tryptase within the gut, extending through the mucosa (MC_T_) into the submucosa and serosa (MC_TC_). These findings within animal models support our clinical observations, with ß-tryptase measurement significantly higher (*P* ≤ 0.001) in the responder group (Fig. 1f). Due to limited availability of biopsy material, we could not investigate whether the immune cells (particularly the MCs) were patient or host derived, but it could be hypothesized that while playing a role in GvHD suppression, these under-investigated immune cells may also support MSC mode of action by controlling inflammation within the local milieu.

In conclusion, we have demonstrated significant differences in the inflammatory milieu of the gut of aGvHD responders and non-responders to MSC therapy. Despite limitations in the analysis we were able to conduct in this study, due to restricted biopsy material taken for diagnostic purposes, we demonstrate that patients who later responded to MSC therapy exhibited an initial gut immune profile with increased MC activity, CD8+ T cells and FoxP3+, and lower levels of CD4+ T cells, CD56+ and CD68+ cells compared to non-responders. These findings suggest that high levels of ongoing inflammation within the gut hinder the therapeutic effect of MSC therapy. Our findings strongly support the need for further validation in a larger cohort of patients. Improvement of biomarkers predicting responsiveness to MSC treatment is of crucial importance for optimal patient treatment, and further understanding regarding both peripheral and tissue-specific immune profiles is required to improve second-line treatments for aGvHD including adoptive MSC transfer.

## Supplementary information

**Additional file 1.** Summary of the statistical analyses using Generalized Estimated Equations with a Poisson distribution in Stata version 14. The CD8, CD4, FOXP3, CD56, CD68 and tryptase tables show the pixel-area predicted incidence rates with 95% confidence intervals. (PDF 203 kb)

## Data Availability

All data generated or analyzed during this study are included in this published article [and its additional file].

## Abbreviations

aGvHD: Acute graft-versus-host disease
aHSCT: Allogeneic hematopoietic stem cell transplantation
APC: Antigen-presenting cell
CMV: Cytomegalovirus
DAB: 3,3-Diaminobenzidine
DLI: Donor lymphocyte infusion
GI: Gastrointestinal
HLA: Human leukocyte antigen
IL: Interleukin
MC: Mast cell
MC_T_: Tryptase-positive mast cell
MC_TC_: Tryptase- and chymase-positive mast cell
MSC: Mesenchymal stromal cell
NA: Not available
NK: Natural killer
RIC: Reduced intensity conditioning
SEM: Standard error of the mean
Tconv: T convertase cell
Th: T helper cell
Treg: T regulatory cell
