# Supplementary material for: Tissue immune profiles supporting response to mesenchymal stromal cell therapy in acute graft-versus-host disease—a gut feeling
Source: Stem Cell Res Ther. 2019 Nov 20;10:334. doi: 10.1186/s13287-019-1449-9 (PMC6864966; doi:10.1186/s13287-019-1449-9)
Supplement: Supplementary file 1 — Additional file 1. Summary of the statistical analyses using Generalized Estimated Equations with a Poisson distribution in Stata version 14. The CD8, CD4, FOXP3, CD56, CD68 and tryptase tables show the pixel-area predicted incidence rates with 95% confidence intervals. (PDF 203 kb) [file 13287_2019_1449_MOESM1_ESM.pdf]

Tissue Immune Profiles Supporting Response to Mesenchymal Stromal Cell Therapy in acute Graft versus Host Disease – a gut feeling

C. Gavin; L. Von Bahr; E. Boberg; M. Bottai; A. Törnqvist Andrén; A. Wernerson; L. C. Davies; R. V. Sugars and K. Le Blanc

Supplementary Data

Summary of the statistical analyses using Generalized Estimated Equations with a Poisson distribution in Stata version 14. The CD8, CD4, FOXP3, CD56, CD68 and tryptase tables show the pixel-area predicted incidence rates with 95% confidence intervals.

Statistical significance was taken with a p-value of <0.05
a = <0.001

Analysis of all images from the 16 patients (8 responders and 8 non-responders).

CD8

CD8	ir	Std. Err.	z	P>|z|	[95% Conf.	Interval]
CD8						
group						
Responders	1.664512	.0060318	140.61	0.000	1.652732	1.676376
_cons	15237.5	43.64273	3362.76	0.000	15152.2	15323.28

Adjusted predictions   Number of obs = 16
Model VCE : OIM

Expression : Predicted incidence rate, predict(ir)

	Margin	Delta-method Std. Err.	z	P>|z|	[95% Conf.	Interval]	
group							
Non-Responders	**15237.5**	43.64273	349.14	0.000	15151.96	15323.04	
Responders	**25363**	56.30608	450.45	0.000	25252.64	25473.36	a.

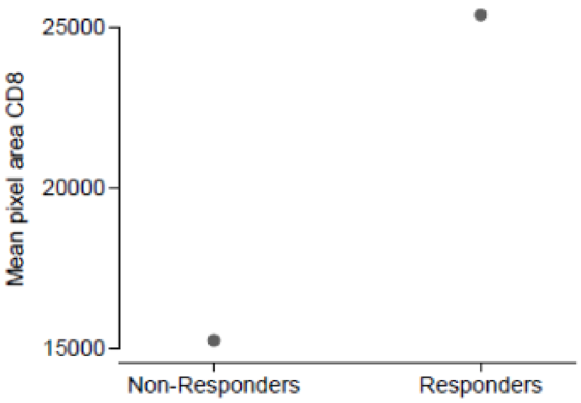

CD4

CD4	ir	Std. Err.	z	P>|z|	[95% Conf.	Interval]
CD4						
group						
Responders	.4805206	.0078048	-45.12	0.000	.4654644	.4960638
_cons	1459.875	13.50868	787.41	0.000	1433.637	1486.593

Adjusted predictions Number of obs = 16
Model VCE : OIM

Expression : Predicted incidence rate, predict(ir)

	Margin	Delta-method Std. Err.	z	P>|z|	[95% Conf.	Interval]	
group							
Non-Responders	**1459.875**	13.50868	108.07	0.000	1433.398	1486.352	
Responders	**701.5**	9.364161	74.91	0.000	683.1466	719.8534	a.

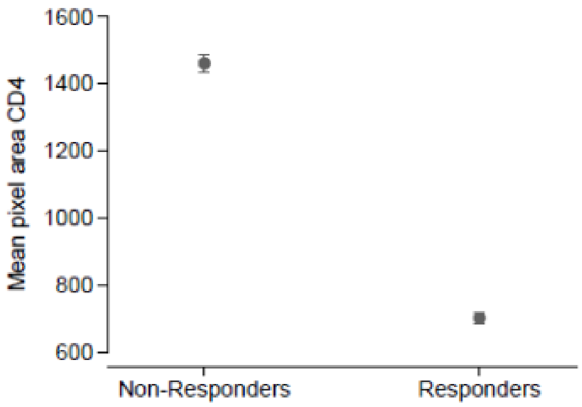

FOXP3

FOXP3	ir	Std. Err.	z	P>|z|	[95% Conf.	Interval]
FOXP3						
group						
Responders	1.381538	.0175151	25.49	0.000	1.347633	1.416297
_cons	1340.625	12.9452	745.74	0.000	1315.491	1366.239

Adjusted predictions Number of obs = 16
Model VCE : OIM

Expression : Predicted incidence rate, predict(ir)

	Margin	Delta-method Std. Err.	z	P>|z|	[95% Conf.	Interval]	
group							
Non-Responders	**1340.625**	12.9452	103.56	0.000	1315.253	1365.997	
Responders	**1852.125**	15.21564	121.73	0.000	1822.303	1881.947	a.

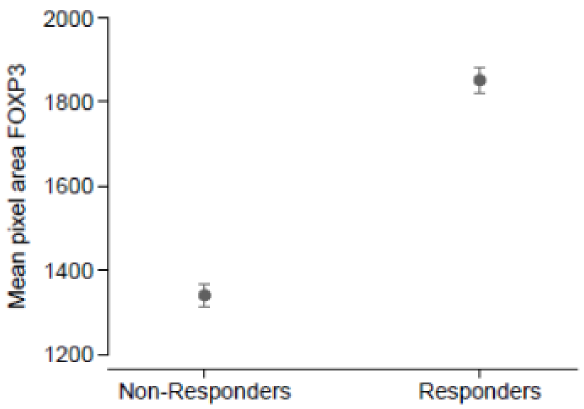

CD56

CD56	ir	Std. Err.	z	P>|z|	[95% Conf.	Interval]
CD56						
group						
Responders	.5969061	.0020127	-153.03	0.000	.5929743	.600864
_cons	29413.13	60.63531	4991.11	0.000	29294.52	29532.21

Adjusted predictions   Number of obs   =   16
Model VCE   : OIM

Expression   : Predicted incidence rate, predict(ir)

	Delta-method Margin	Std. Err.	z	P>|z|	[95% Conf.	Interval]
group						
Non-Responders	**29413.13**	60.63531	485.08	0.000	29294.28	29531.97
Responders	**17556.88**	46.84666	374.77	0.000	17465.06	17648.69

a.

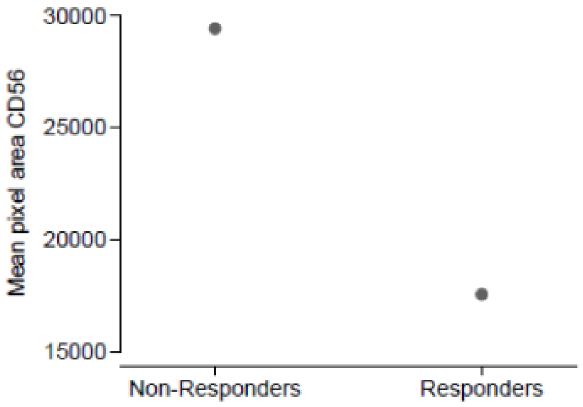

CD68

CD68	ir	Std. Err.	z	P>|z|	[95% Conf.	Interval]
CD68						
group						
Responders	.8210743	.0018596	-87.04	0.000	.8174376	.8247272
_cons	54046.88	82.19403	7165.75	0.000	53886.02	54208.21

```
Adjusted predictions                            Number of obs     =         16
Model VCE    : OIM

Expression   : Predicted incidence rate, predict(ir)
```

	Delta-method Margin	Std. Err.	z	P>|z|	[95% Conf.	Interval]	
group							
Non-Responders	**54046.88**	82.19403	657.55	0.000	53885.78	54207.97	
Responders	**44376.5**	74.4786	595.83	0.000	44230.52	44522.48	a.

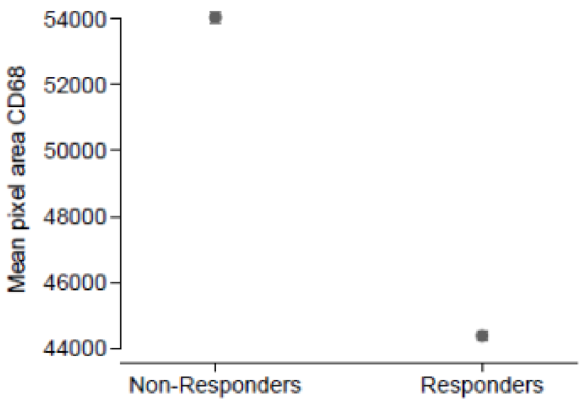

Tryptase

Tryptase	ir	Std. Err.	z	P>|z|	[95% Conf.	Interval]
Tryptase						
group						
Responders	2.065573	.0055334	270.79	0.000	2.054757	2.076447
_cons	25850.75	56.84491	4620.40	0.000	25739.58	25962.4

```
Adjusted predictions                            Number of obs     =         16
Model VCE    : OIM

Expression   : Predicted incidence rate, predict(ir)
```

	Delta-method Margin	Std. Err.	z	P>|z|	[95% Conf.	Interval]	
group							
Non-Responders	**25850.75**	56.84491	454.76	0.000	25739.34	25962.16	
Responders	**53396.63**	81.69809	653.58	0.000	53236.5	53556.75	a.

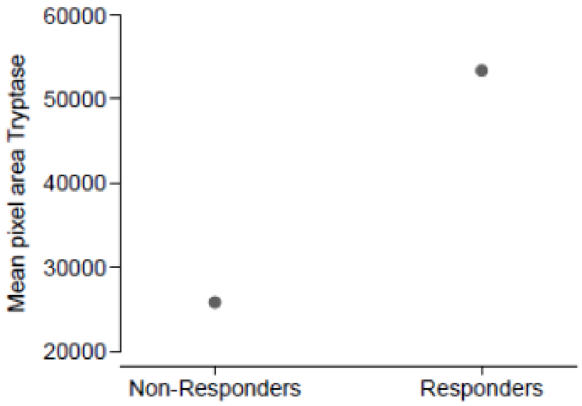
60000
50000
40000
30000
20000
Mean pixel area Tryptase
Non-Responders
Responders
